# A rare case of hemolytic-uremic syndrome due to a *Capnocytophaga canimorsus* sepsis: Case report

**DOI:** 10.1097/MD.0000000000045266

**Published:** 2025-10-24

**Authors:** Pierre-Alexis Tschuy, Nicerine Krause, Jacques Serratrice, Matteo Coen

**Affiliations:** a Department of Medicine, Service of General Internal Medicine, Geneva University Hospitals, Geneva, Switzerland; b Department of Surgery, Service of Transplantation and Visceral Surgery, Geneva University Hospitals, Geneva, Switzerland; c Faculty of Medicine, Unit of Development and Research in Medical Education (UDREM), University of Geneva, Geneva, Switzerland.

**Keywords:** *Capnocytophaga canimorsus*, dog exposure, hemolytic-uremic syndrome, sepsis, thrombotic microangiopathy

## Abstract

**Rationale:**

*Capnocytophaga canimorsus* infection is a rare complication of pet exposure, often resulting from a bite or lick. Its prolonged bacterial culture time can delay diagnosis and treatment, potentially leading to life-threatening situations, such as thrombotic microangiopathy (TMA), especially in immunocompromised patients.

**Patient concerns:**

A 62-year-old immunocompetent man was admitted with septic shock.

**Diagnoses:**

During hospitalization, he developed hemolytic-uremic syndrome with anuric acute kidney injury. Blood cultures later confirmed *C canimorsus* bacteremia. On reassessment, he recalled a recent index-finger skin injury that had been licked by his dog.

**Interventions:**

The patient received hemodialysis and antibiotic therapy with amoxicillin–clavulanate.

**Outcomes:**

He achieved full clinical recovery.

**Lessons:**

TMA secondary to *C canimorsus* bacteremia is exceptionally rare (<20 cases reported). Because the organism grows slowly, clinicians should maintain a high index of suspicion in septic patients with recent dog exposure – especially when TMA is present on admission – and start appropriate empiric therapy while cultures are pending. Early distinction between primary thrombotic thrombocytopenic purpura/hemolytic-uremic syndrome and infection-associated TMA is essential to guide management.

## 1. Introduction

A 62-year-old immunocompetent man, suffering from hypertension, ischemic heart disease and a previously diagnosed vestibular schwannoma, presented fever and a painful swelling of his left index. His general practitioner suspected gout. Initial management consisted of colchicine 0.5 mg 2 times a day for 48 hours. The patient developed diarrhea and vomiting, leading to his admission to our emergency room department the following day.

**Figure 1.**
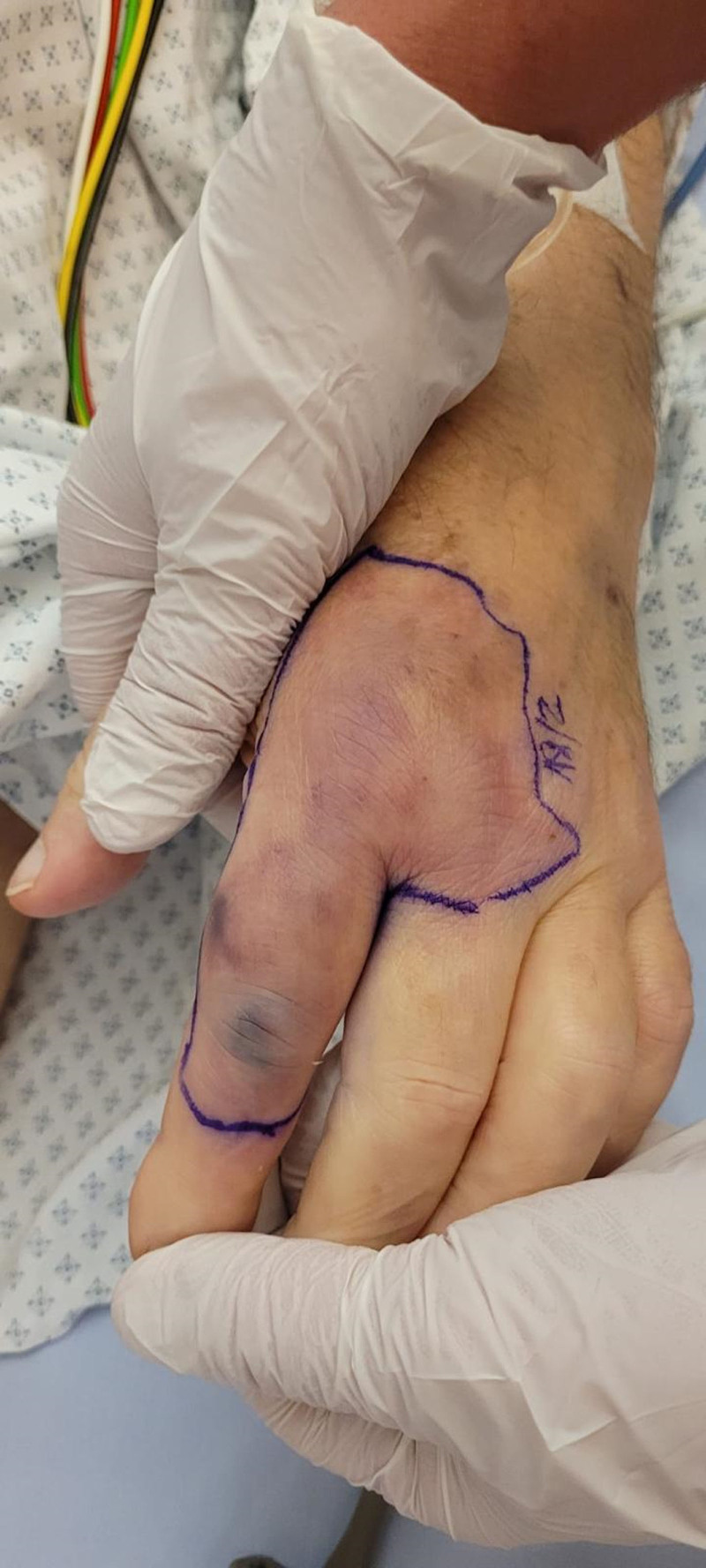
Erythema of the left index 7 days after admission.

**Figure 2.**
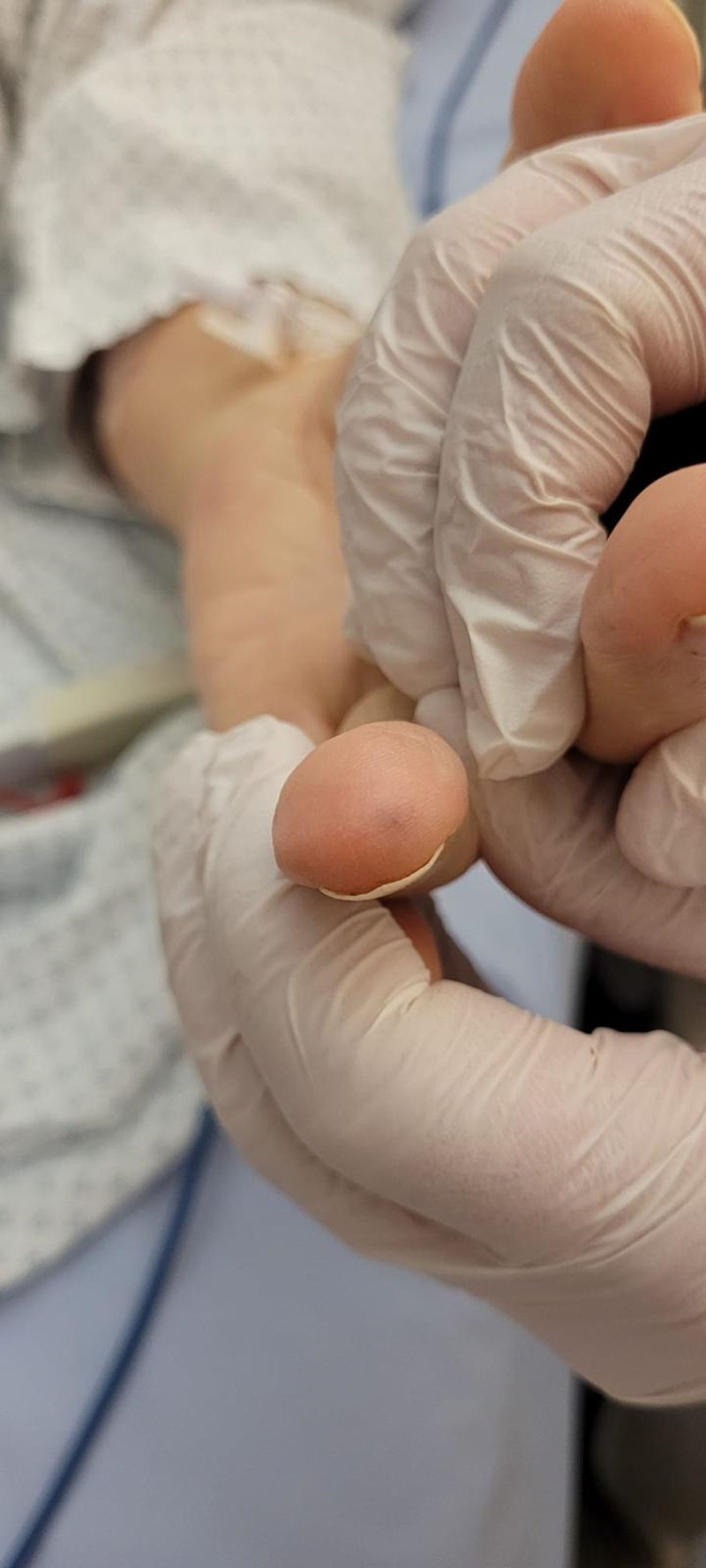
Left punctiform lesion corresponding to the inial injury.

**Figure 3.**
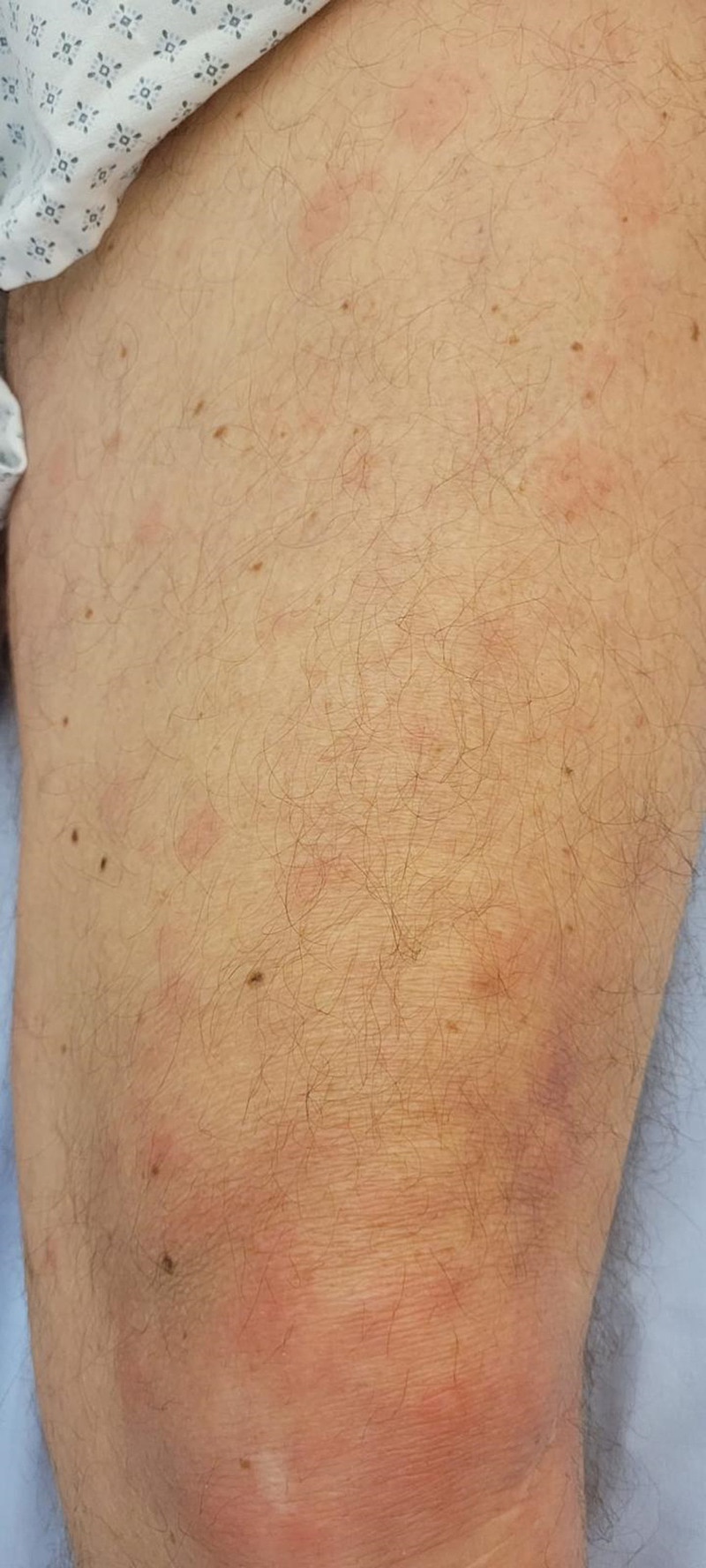
Initial polymorphous rash.

Upon admission, he exhibited signs of sepsis, with hypotension and marbling alongside a non-acute diffusely tender abdomen. The left index finger was painful, without swelling or erythema. Additionally, a polymorphous erythema was also noted on the torso and legs (Figs. 1–3). Laboratory findings indicated a significant systemic inflammation with a C-reactive protein measured at 300mg/l, stage 1 renal insufficiency according to KDIGO criteria, and mild thrombocytopenia. (Table 1). Chest X-ray was unremarkable, and abdominopelvic CT scan was normal. A digestive source of infection was first suspected and prompted an empirical treatment with Ceftriaxone and Metronidazole. The patient’s condition worsened and progressed to septic shock requiring transfer to the intensive care unit. Laboratory tests revealed a normochromic, normocytic, regenerative anemia with a hemoglobin level as low as 80 g/L, a reticulocyte production index higher than 3 (5.3), elevated levels of bilirubin (54 µmol/L) and lactate dehydrogenase (1283 U/L), as well as a diminished Haptoglobin at 189 mg/L, indicating hemolytic anemia. Present also was severe thrombocytopenia with a platelet count of 8 G/L (Table 1). Blood smear showing schistocytes pointed towards a diagnosis of thrombotic microangiopathy. Due to the possibility of Thrombotic thrombocytopenic purpura (TTP), initial empiric treatment consisted of a single dose of Aplacizumab and plasmapheresis. The antibiotic coverage was broadened to Piperacillin/Tazobactam.

**Table 1:** Laboratory values during hospitalization.

	On admission (February 17, 2023)	On diagnosis of TTP (February 20, 2023)	At discharge after supportive treatment (March 14, 2023)
Hemoglobin (g/L)	133	80	100
White blood cells (G/L)	3.9	15.7	8.3
Platelets (G/L)	137	5	477
BUN (mmol/L)	10	25.8	7.5
Creatinine (µmol/L)	109	455	122
Total bilirubin (µmol/L)	21	54	5
PT (%)	65	89	100
PTT	33.7	48.6	31.6
INR	1.22	1.04	1
Fibrinogen			
Lactate dehydrogenase (U/L)		1283	534
ADAMTS13 activity (%)		22	

ADAMTS13 = a disintegrin and metalloprotease with thrombospondin type 1 motif, member 13.

ADAMTS13 activity was measured at 22% (N >20%), leading to the diagnosis of hemolytic-uremic syndrome (HUS) while tests for Shiga toxins were negative. Due to the exclusion of TTP, plasmapheresis and Aplacizumab was not pursued.

Renal function worsened to anuria and continuous hemodialysis was initiated. Subsequent blood cultures identified *Capnocytophaga Canimorsus*, with a broad sensitivity, notably to Amoxicillin and Clavulanic Acid. Echocardiography showed no evidence of endocarditis, enabling us to narrow antibiotic treatment to Amoxicillin and Clavulanic Acid with a progressive improvement of the patient’s condition.

Considering the results of blood cultures, a detailed reexamining of the patient’s history revealed exposure to his dog’s saliva, which had licked an open wound on his index finger a week prior, pinpointing the source of infection. The initial polymorphous erythema (Fig. 1) and the gastrointestinal symptoms were subsequently attributed to colchicine side effects. When seen 3 months later, the patient achieved complete recovery, with resolution of the thrombotic microangiopathy and renal insufficiency.

## 2. Discussion

We report the case of an immunocompetent patient who suffered severe HUS due to *Capnocytophaga canimorsus* infection contracted from a wound licked by his own dog. *Capnocytophaga canimorsus* is an anaerobic gram-negative encapsulated bacillus, from the *Falvobacteriaceae family.*^[1]^ This commensal organism is found in the oral flora of many canine and feline species that can occasionally infect humans, usually after bites or contact of wounds with animal saliva. The incubation period varies from 1 to 8 days, but most patients show initial symptoms within 48 hours after exposure. Symptoms are generally benign and nonspecific, often mimicking a viral infection, and consist of nausea, vomiting, diarrhea, fever, dyspnea and exanthema. *Capnocytophaga canimorsus* is known for its slow culture growth, which can lead to diagnostic delays. It is most sensible to amoxicillin and clavulanic acid. The initial symptoms of this illness are often nonspecific. Also, patients may not realize that their symptoms could be related to a past instance of being licked by an animal, as they may have had limited contact with animal saliva. Consequently, they may fail to mention this during medical consultations.^[2]^ This underreporting, along with the slow growth of cultures, can significantly delay the diagnosis, potentially resulting in misdiagnosis and inappropriate treatment.^[3]^

Patients with asplenia, under immunosuppressive medication, or with chronic alcohol consumption present a higher risk of sepsis. Necrotizing fasciitis, meningitis, endocarditis, and Disseminated Intravascular Coagulation can also complicate the course of this infection.^[4]^ Our patient was immunocompetent, thus the initial use of colchicine in his case could have influenced the disease course, by altering the chemotaxis of cells, ultimately facilitating sepsis.

Among bleeding complications, thrombotic micro-angiopathy is one of the rarest but most severe complications of *Capnocytophaga canimorsus* infection.^[5]^ ADAMTS 13 activity was measured at 23% in our patient, this point is crucial for differentiating HUS from TTP, it remains within the normal range in HUS (above 20%) and is significantly reduced (below 10%) in TTP. As the delivery of the result of ADAMTS13 activity in our patient was delayed, we initially introduced Caplacizumab and plasmapheresis, however, treatment for HUS primarily involves supportive care, including hemodialysis if necessary, and specific antibiotic therapy, whereas plasmapheresis is usually not effective.^[6]^ Understanding the distinctions between these 2 conditions is crucial, especially in cases of *Capnocytophaga canimorsus* sepsis, where the onset of thrombotic microangiopathy requiring immediate intervention may occur before bacteriological culture results are available.

## 3. Conclusion

To date, only 12 cases^[7–18]^ of thrombotic micro-angiopathy are described in the literature (Table 2), mostly HUS. Only one case of severe TTP due to *Capnoytophaga canimorsus* infection is reported, in which platelet count and ADAMTS13 activity normalized after amoxicillin-clavulanic acid treatment alone. In our case, the patient was an immunocompetent patient with a little wound simply licked by his dog. To our point of view, the role of colchicine in triggering the onset of sepsis and ultimately HUS, is questioning.

**Table 2:** Review of case report of *Capnocytophaga canimorsus* associated with thrombotic thrombocytopenic purpura and/or hemolytic-uremic syndrome.

References	Year	Animal exposure	Age and gender	Symptoms/signs	Co-morbidities	ADAMS13 Activity	HUS/TTP	Treatment
Naoussi et al^[7]^	2022	Bitten in the hand by her own dog	42 yr of female	Headaches, myalgias, abdominal pain and punctiform lesion in the right hand	Asthma	44%	Secondary SHU	Amoxicillin acid clavulanic, Plasmaphereses
Tani et al^[8]^	2019	Bitten by her own dog	62 yr of female	Fever, abdominal pain and diarrhea	No history	77.2 %	TMA	Piperacillin and tazobactam, Thrombomodulin alfa, Platelet transfusion, Plasma exchange, Ampicillin and amoxicillin
Smeets et al^[9]^	2018	No exposure	63 yr of male	Diarrhea, confusion, general weakness	Alcoholism, Hypertension, Pulmonary emphysema, Myocardial infarction with cardioverter defibrillator	<1%	Secondary TMA	Ceftriaxone, Thiamine, Plasma Infusion,
Sokol et Al^[10]^	2016	No exposure	31 yr of male	Altered mental status, witnessed seizure, circulatory shock associated with organ failures	No history	NA	Atypical HUS	Antibiotics, Eculizumab
Maezawa et al^[11]^	2016	Bitten in the hand by his own dog	61 yr of male	High fever, black wound in the left finger from bites	No history	39 %	Secondary TMA	Meropenem, Clindamycin, Hemofiltration, Mechanical ventilation support, Plasma Exchange
Ma et al^[12]^	2013	Bitten in the face by his own dog bite	56 yr of male	Fatigue, gait instability, nausea.	Traumatic splenectomy 22 years ago	NA	Features of TTP without dysregulation of coagulation	Vancomycin, Piperacillin and Tazobactam Ampicillin-sulbactam, Prednisone, Plasma Exchange
Brichacek et al^[13]^	2012	Cut in hand dog licked wound	72 yr of male	Abdominal pain, nausea, vomiting, diarrhea and confusion	Ribs fractures after a fall 4 months ago	NA	TTP	Plasma exchange, Piperacillin-Tazobactam Imipenem, Meropenem
Mulder et al^[14]^	2001	Dog bite	66 yr of male	Fever, malaise, chills, vomiting diarrhea, hematemesis	Hypertension, Diabetes, hypercholesterolemia, AIT	NA	HUS	Cefuroxime, metronidazole, amoxicillin/clavulanate, plasmapheresis, hemodialysis
Kok et al^[15]^	1999	No history of animal attack but owns a dog	47 yr of male	Fever icterus, abdominal pain, dyspnea, hematemesis, melena	Alcoholism	NA	TTP	Amoxicillin/clavulanate, Ofloxacin, Plasma exchange, Hemodiafiltration
Tobe et al^[16]^	1999	Bitten in the hand by his own dog	50 yr of male	Healthy	Fever, arthralgia, oliguria, small puncture on his left hand	NA	HUS	Amoxicillin/clavulanate, Plasma exchange.
Finn et al^[17]^	1996	Owned dog	53 yr of female	Fever, headache, arthralgia, confision, purpura	Tobocco use	NA	TTP	Benzylpenicillin, High-dose steroids, Plasma exchange, Hemofiltration
Scarlett et al^[18]^	1990	Own cat scratches	72 yr of male	Fever malaise, rigors, dyspnea, blindness of right eye, purpura and melena	Healthy	NA	TTP	Gentamicin, Flucloxacillin, Penicillin, Prednisolone, Plasma exchange, Imipenem
Scarlett et al^[18]^	1990	No animal contact	49 yr of male	Fever, vomiting diarrhea, rigors, arthralgia, cough, swollen painful blue blotchy fingers and toes	Splenectomy 15 yr ago	NA	TTP	Amoxicillin/clavulanate, ofloxacin, plasma exchange, hemodiafiltration

ADAMTS13 = a disintegrin and metalloprotease with thrombospondin type 1 motif, member 13, HUS = hemolytic-uremic syndrome, TMA = thrombotic microangiopathy, TTP = thrombotic thrombocytopenic purpura.

The slow growth rate of the bacterium poses significant diagnostic challenges, particularly in severe cases where microangiopathy is present at admission. This underscores the importance of distinguishing between the primary types of thrombotic microangiopathy and highlights the effective treatment protocol for such infections and recalls that the simple lick of a dog can lead to severe conditions.

## Author contributions

**Conceptualization:** Pierre-Alexis Tschuy, Jacques Serratrice, Matteo Coen.

**Supervision:** Matteo Coen.

**Visualization:** Nicerine Krause.

**Writing – original draft:** Pierre-Alexis Tschuy, Matteo Coen.

**Writing – review & editing:** Pierre-Alexis Tschuy, Nicerine Krause, Jacques Serratrice, Matteo Coen.
